# Supplementary material for: Empirical methods for controlling false positives and estimating confidence in ChIP-Seq peaks
Source: BMC Bioinformatics. 2008 Dec 5;9:523. doi: 10.1186/1471-2105-9-523 (PMC2628906; doi:10.1186/1471-2105-9-523)
Supplement: Additional File 1 — A variety of html documents from the USeq web site detailing the available applications, their best usage, output file type descriptions, command line menus, etc. [file 1471-2105-9-523-S1.zip › USeqUserGuides/JavaDoc.zip!/JavaDoc/edu/utah/seq/methylation454/class-use/Sample.html]

Uses of Class edu.utah.seq.methylation454.Sample


|  |  |  |
| --- | --- | --- |
| **Overview** \| **Package** \| **Class** \| **Use** \| **Tree** \| **Deprecated** \| **Index** \| **Help** | |  |
| PREV   NEXT | **FRAMES**    **NO FRAMES**     **All Classes** |  |


---


## **Uses of Class edu.utah.seq.methylation454.Sample**

| Packages that use Sample | |
| --- | --- |
| **edu.utah.seq.methylation454** | Applications for working with 454 data and identifying bisulfite converted bases for methylation studies. |

Uses of Sample in edu.utah.seq.methylation454

| Methods in edu.utah.seq.methylation454 that return Sample | |
| --- | --- |
| `Sample[]` | `MethylationParser.getSamples()` |

| Methods in edu.utah.seq.methylation454 with parameters of type Sample | |
| --- | --- |
| `static java.util.ArrayList` | `StatMethylationData.findCommonAmplicons(Sample one, Sample two)` |
| `void` | `StatMethylationData.statCpGs(Sample one, Sample two)`             Generates two tailed Chi-square p-values for differences in %C (a proxy for %methylation) for each CpG in each Amplicon between two samples and a Wilcoxon Signed Rank p-value for all of the CpG %Cs in an amplicon. |

---


|  |  |  |
| --- | --- | --- |
| **Overview** \| **Package** \| **Class** \| **Use** \| **Tree** \| **Deprecated** \| **Index** \| **Help** | |  |
| PREV   NEXT | **FRAMES**    **NO FRAMES**     **All Classes** |  |


---
